# Chromosomal mapping of rRNA genes, core histone genes and telomeric sequences in *Brachidontes puniceus *and *Brachidontes rodriguezi *(Bivalvia, Mytilidae)

**DOI:** 10.1186/1471-2156-11-109

**Published:** 2010-12-10

**Authors:** Concepción Pérez-García, Jorge Guerra-Varela, Paloma Morán, Juan J Pasantes

**Affiliations:** 1 Dpto. Bioquímica, Xenética e Inmunoloxía. Universidade de Vigo. Vigo, Spain

## Abstract

**Background:**

Chromosome rearrangements are an important part of the speciation process in many taxa. The study of chromosome evolution in bivalves is hampered by the absence of clear chromosomal banding patterns and the similarity in both chromosome size and morphology. For this reason, obtaining good chromosome markers is essential for reliable karyotypic comparisons. To begin this task, the chromosomes of the mussels *Brachidontes puniceus *and *B. rodriguezi *were studied by means of fluorochrome staining and fluorescent *in situ *hybridization (FISH).

**Results:**

*Brachidontes puniceus *and *B. rodriguezi *both have 2n = 32 chromosomes but differing karyotype composition. Vertebrate-type telomeric sequences appear at both ends of every single chromosome. *B. puniceus *presents a single terminal major rRNA gene cluster on a chromosome pair while *B. rodriguezi *shows two. Both mussels present two 5S rDNA and two core histone gene clusters intercalary located on the long arms of two chromosome pairs. Double and triple-FISH experiments demonstrated that one of the 5S rDNA and one of the major rDNA clusters appear on the same chromosome pair in *B. rodriguezi *but not in *B. puniceus*. On the other hand, the second 5S rDNA cluster is located in one of the chromosome pairs also bearing one of the core histone gene clusters in the two mussel species.

**Conclusion:**

Knowledge of the chromosomal distribution of these sequences in the two species of *Brachidontes *is a first step in the understanding of the role of chromosome changes on bivalve evolution.

## Background

Over recent years, an increasing number of molecular studies have focused on the elucidation of the phylogenetic relationships among species of bivalves in order to further reveal their evolutionary history [1]. Nevertheless, cytogenetic comparisons between species remain quite rare mainly due to difficulties in obtaining good chromosome banding patterns [2]. This fact, together with the small differences in chromosome size and morphology in many species of bivalves, makes the unequivocal identification of each chromosome pair very difficult and therefore, complicates cytogenetic comparisons.

Fluorescent *in situ *hybridization (FISH) allows combining cytogenetic and molecular techniques, improving our understanding of genome organization. The chromosomal mapping of different types of DNA sequences in bivalves has greatly increased over the last years [2-4]. Although the information gained so far is of great importance in the analysis of chromosome evolution in bivalves, the data obtained are still scarce and focused on a limited number of DNA sequences, mainly in species with commercial interest.

Most FISH reports in bivalves have been concentrated on the chromosomal localization of major rRNA genes rather than on other gene families such as 5S rDNAs and histone genes [2,5]. Major rDNA has been located in about 50 species of bivalves [2,5], including seven species of the family Mytilidae [5-11]. 5S rDNA has been located in a small number of species belonging to the families Cardiidae [12], Mytilidae [5,11], Ostreidae [13,14] and Pectinidae [15-20]. The organization of the histone genes has been characterized in two species of mussels [21-24], one scallop [25] and three species of venerid clams [26] but their chromosomal locations are only known in two species of mussels [5,22,24] and four species of Pectinidae [27]. Telomeric sequences have been characterized and located in a few species of bivalves, including two species of Mytilidae [5,6,28].

*Brachidontes *(*senegalensis*) *puniceus *(Gmelin 1791) is a small mussel species native to the African Atlantic coast, from Mauritania to Angola. *B. rodriguezi *(d'Orgbigny 1846) is a medium-sized mussel distributed from Río Grande do Sul (Brazil) to the northern part of Patagonia (Argentina) on the Atlantic coast of South America. Karyological data on the genus *Brachidontes *is limited to the knowledge of the karyotypes and the chromosomal location of the major rDNA in *B. rodriguezi *[8] and *B. pharaonis *[10]. In order to expand the cytogenetic information on the family Mytilidae, we analyzed the chromosomes of *B. puniceus *and *B. rodriguezi *by means of 4',6-diamidino-2-phenylindole (DAPI)/propidium iodide (PI) and chromomycin A3 (CMA)/DAPI fluorescence staining and fluorescent *in situ *hybridization (FISH) with 28S rDNA, 5S rDNA, core histone genes and telomeric probes.

## Methods

### Animals

Samples of *Brachidontes puniceus *were collected at São Vicente (Cape Verde) and São Tomé (São Tomé and Principe). *B. rodriguezi *specimens were collected at Mar del Plata (Argentina). Mussels were maintained in the laboratory in tanks of 5 L of aerated, filtered seawater at 18 ± 1°C and fed on a mixture of microalgae cells (*Isochrysis galbana *and *Tetraselmis suecica*) for at least 15 days in order to promote both somatic growth and gonadic maturation.

### Chromosome preparation and fluorochrome staining

Chromosome preparations were obtained following the technique described previously [6]. Juvenile specimens were housed in 0.5 L beakers and exposed to colchicine (0.005%) for 12 h. Gill and mantle tissues were excised and immersed in 50% and 25% seawater for 90 min and fixed with ethanol/acetic acid for 1 h. Chromosome spreads were obtained by dissociating small pieces of tissue in 60% acetic acid, pipetting suspension drops onto slides heated to 55°C and air-drying.

Some chromosome preparations were stained for 2 h with CMA (0.25 mg mL^-1^) and counterstained with DAPI (0.14 μg mL^-1^) for 8 min. Once washed with distilled water, slides were air-dried and mounted with antifade (Vectashield, Vector). After visualization and photography, preparations were washed and re-stained with a combination of DAPI (0.14 μg mL^-1^) and PI (0.07 μg mL^-1^). The slides were then washed in distilled water, air-dried, mounted in antifade and photographed again.

Chromosome counts and karyotype analysis were performed in 10 specimens (5 males, 5 females) of each species. In order to determine the karyotype of *Brachidontes puniceus*, ten good metaphase plates were used to construct karyotypes. Chromosome and arm lengths were carefully measured and relative lengths and centromeric indices were calculated. Chromosome nomenclature follows Levan et al. [29].

### DNA extraction, PCR amplification and probe labeling

Total DNA was extracted following the method of Estoup et al. [30] with minor modifications. About 3 mg of adductor muscle tissue was homogenized in 0.4 mL of a pre-warmed (60°C) 10% Chelex 100 (BioRad) solution. After adding pronase (1.4 mg mL^-1^) and incubating for 1 h at 60°C in agitation, the extracted DNA was stored at 4°C.

FISH probes were obtained by PCR. Amplifications were performed in 20 μl of a solution containing 50 ng DNA, 1× PCR buffer, 0.5 mM each dNTP, 2.5 mM MgCl_2_, 1 μM each primer and 1 U BIOTAQ DNA polymerase (Bioline). The combinations of primers used in the amplification appear in Table 1. Universal primers (*LR10R, LR12*) were used to amplify a fragment of the 28S rRNA gene of the major rDNA repeat [31]. For the 5S rDNA amplification, primers were designed from the sequence of the 5S rRNA of *Mytilus edulis *[32]. The amplifications of the H2B/H2A and the H3 histone genes were performed using primers designed from the sequences of the histone genes of *Mytilus edulis *[23] and those described by Giribet and Distel [1], respectively. After 5 min denaturation at 95°C, 30 cycles of amplification were performed using the conditions that are shown in Table 1. A final extension step of 7 min at 72°C was applied. All reactions were performed in a GeneAmp PCR system 9700 (Applied Biosystem) and PCR products were examined by electrophoresis on a 2% agarose gel. Single products were obtained after amplification using each set of primers. 28S rDNA was labeled with biotin 16-UTP and/or digoxigenin-UTP (10× DIG Labeling Mix, Roche) by nick translation. 5S rDNA and histone genes were directly labeled by PCR either with biotin-16-UTP or digoxigenin-UTP. The labeled PCR products were precipitated before FISH.

**Table 1:** Primers and parameters used in the PCR

Gene	Sequences of the primers	Denaturation	Annealing	Elongation
*28S*	LR10R: 5'GACCCTGTTGAGCTTGA3' LR12: 5'GACTTAGAGGCGTTCAG3'	95°C, 20 s	48°C, 20 s	72°C, 30 s
*5S*	F: 5'CAACGTGATATGGTCGTAGAC3' R: 5'AACACCGGTTCTCGTCCGATC3'	95°C, 30 s	44°C, 30 s	72°C, 30 s
*h2ba*	F: 5'TCCCGAGATGTGATGGTAGA3' R: 5'AGTACAGCCTGGATGTTTGGTAA3'	95°C, 30 s	45°C, 30 s	72°C, 40 s
*h3*	F: 5'ATGGCTCGTACCAAGCAGACVGC3' R: 5'ATATCCTTRGGCATRATRGTGAC3'	95°C, 15 s	48°C, 15 s	72°C, 15 s

### Fluorescent *in situ *hybridization (FISH)

Single and double FISH experiments were performed following the methods published previously [33] with minor modifications. Preparations were denatured at 69°C for 2 min and hybridized overnight at 37°C. Signal detection was carried out with fluorescein avidin and biotinylated anti-avidin for the biotinylated probes and with mouse antidigoxigenin, goat anti-mouse rhodamine and rabbit anti-goat rhodamine for the digoxigenin-labeled probes. Slides were counterstained with DAPI and mounted in antifade.

In order to map three probes on the same metaphase plates, two consecutive FISH were performed. In the first one, a histone gene probe labeled with biotin and a 5S rDNA probe labeled with digoxigenin were employed. After visualization and photography, the preparations were re-hybridized using a 28S rDNA probe simultaneously labeled by nick translation with both biotin and digoxigenin and the same metaphase plates were visualized and photographed again. In addition, we also performed FISH with a telomeric (CCCTAA)_3 _peptide nucleic acid (PNA) probe (Applied Biosystems) following the protocol indicated by the supplier.

Slide visualization and photography were performed with a Nikon Eclipse-800 microscope equipped with an epifluorescence system. Separated images for each fluorochrome were obtained with a Sensys CCD camera (Photometrics) connected to the microscope. Camera control and digital image acquisition employed a Power Macintosh computer. Pseudocoloring and merging of the images was performed with Adobe Photoshop.

## Results

**Figure 1:**
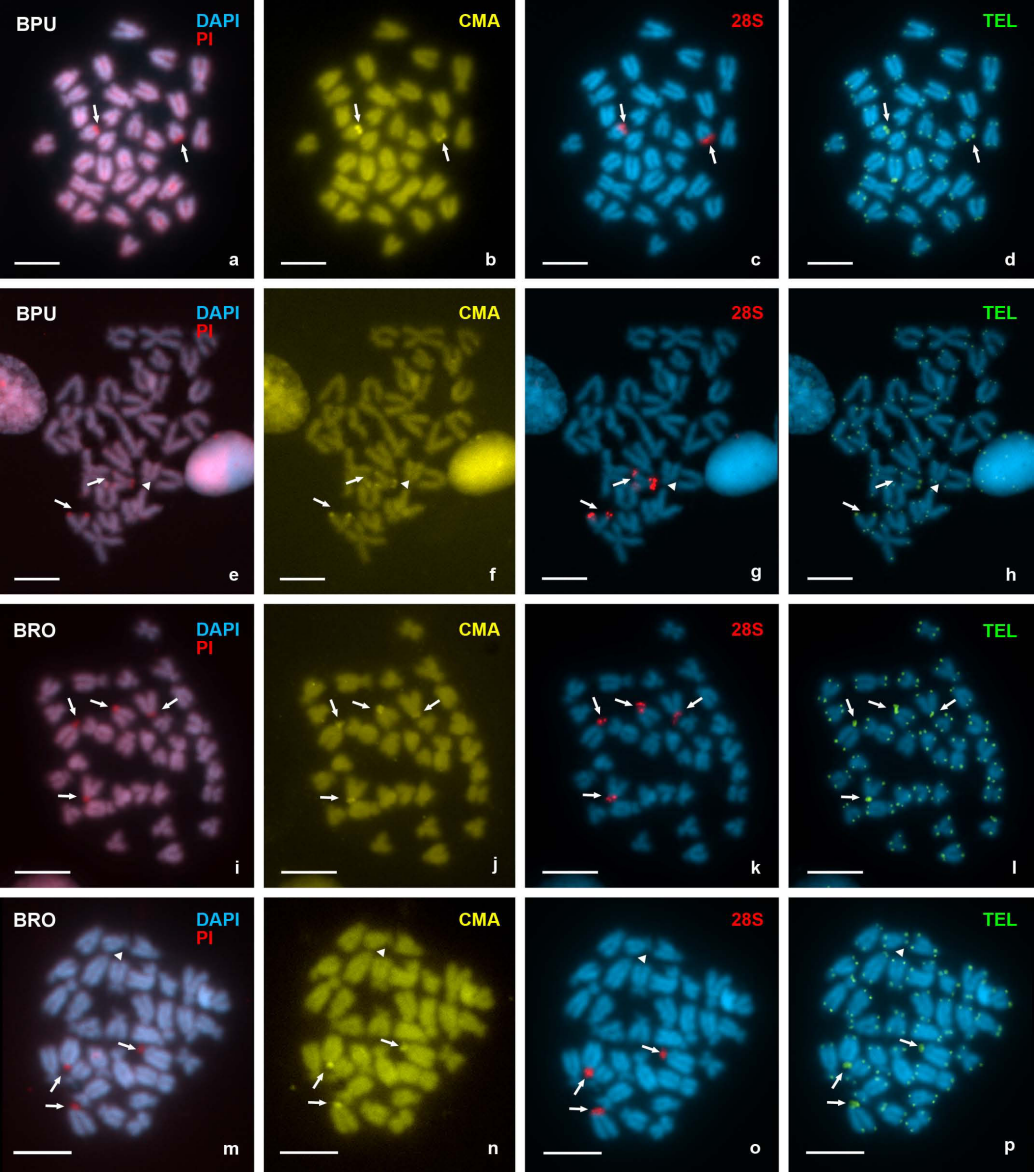
**Sequential DAPI/PI and CMA staining followed by FISH using 28S rDNA (28S) and telomeric (TEL) probes on *Brachidontes puniceus *(BPU) and *Brachidontes rodriguezi *(BRO) chromosomes**. Sequential staining of the same metaphase plates with DAPI/PI (**a, e**) and CMA (**b, f**) shows the presence of two GC-rich (DAPI dull/CMA bright) regions located at the ends of the long arms on one chromosome pair in *B. puniceus *(arrows in **a**, **b**, **e**, **f**). One of the *B. puniceus *specimens shows an additional GC-rich region on the short arm of one of the members of the chromosome pair bearing the standard band (arrowhead in **e**, **f**). In *B. rodriguezi *four GC-rich regions appear on the short arms of two chromosome pairs (arrows in **i**, **j**) but one of the mussels shows only three of these bands (arrows in **m**, **n**) lacking the fourth (arrowhead in **m**, **n**). FISH using a 28S rDNA probe (digoxigenin, rhodamine, red) gives signals at the CMA+ positions in both *B. puniceus *(arrows in **c**, **g**, arrowhead in **g**) and *B. rodriguezi *(arrows in **k**, **o**). Note the absence of the fourth major rDNA signal in **o **(arrowhead). FISH using a PNA-telomeric probe (fluorescein, green) gives signals at both ends of every sister chromatid. Telomeric signals situated on the GC-rich NORs are consistently bigger than the rest of the telomeric signals and their sizes are similar to the sizes of the 28S signals (arrows in **d, h, l, p**). Scale bars, 5 μm.

**Figure 2:**
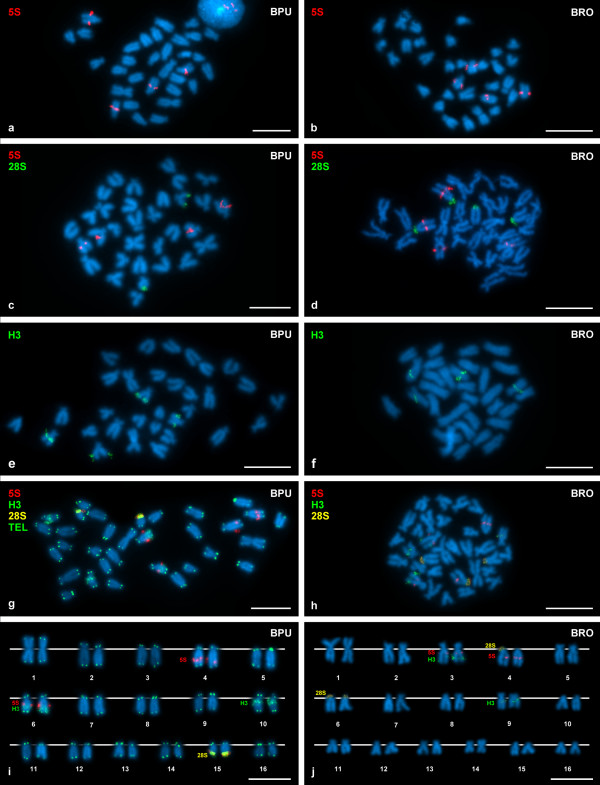
**Chromosomal location of 5S rDNA, major rDNA and core histone genes on *Brachidontes puniceus *and *Brachidontes rodriguezi***. FISH using a 5S rDNA probe (digoxigenin, rhodamine, red) reveals the presence of two clusters of 5S rDNA on two chromosome pairs in both *B. puniceus *(BPU, **a**) and *B. rodriguezi *(BRO, **b**). Double-FISH experiments using a 5S rDNA probe (digoxigenin, rhodamine, red) and a 28S rDNA probe (biotin, fluorescein, green) show that major and minor gene clusters in *B. puniceus *(**c**) are to be found on different chromosome pairs but that in *B. rodriguezi *(**d**) one of the chromosome pairs bearing a 5S rDNA signal also bears one of the major rDNA signals. FISH using a histone H3 gene probe (biotin, fluorescein, green) shows two clusters of core histone genes on the long arms of two chromosome pairs in both *B. puniceus *(**e**) and *B. rodriguezi *(**f**). Triple hybridization (**g**, **h**) using a digoxigenin labeled 5S rDNA probe (red), a biotin labeled histone H3 gene probe (red) and a double (biotin and digoxigenin) labeled 28S rDNA probe (yellow, as a result of the mixture of green and red) reveals that the histone gene clusters are independent of the major rDNA clusters in *B. puniceus *(**g**, **i**) and in *B. rodriguezi *(**h**, **j**). In both species, one of the chromosome pairs bearing a 5S rDNA signal also carries one of the core histone gene signals (**g**, **h**, **i, j**). The metaphase plate in **g**, **i **was also re-hybridized using a PNA telomeric probe (fluorescein, green). Scale bars, 5 μm.

Coinciding with previously reported data [8], *Brachidontes rodriguezi *presents a diploid chromosome number of 2n = 32 (Figure 1i-p) and its karyotype is composed of 2 metacentric, 12 subtelocentric and 2 telo/subtelocentric chromosome pairs (Figure 2j). *B. puniceus *also shows a diploid complement of 2n = 32 chromosomes (Figure 1a-h). The karyotype is composed of 1 metacentric, 3 submetacentric, 4 submeta/subtelocentric, 4 subtelocentric and 4 telocentric chromosome pairs (Figure 2i). A summary of the results obtained after measuring the DAPI-stained chromosomes of 10 of the best complete metaphase plates is given in Table 2. No differences were observed between the São Vicente and São Tomé populations.

**Table 2:** Relative lengths and centromeric indices of Brachidontes puniceus chromosomes

Pair	Relative length		Centromeric index		Type
		
	Mean	SE	Mean	SE	
1	8.72	0.53	43.48	0.78	m
2	7.99	0.50	25.48	1.09	sm/st
3	7.20	0.27	16.21	0.90	st
4	7.05	0.34	20.56	0.77	st
5	6.96	0.54	8.59	0.55	t
6	6.56	0.40	24.66	1.20	sm/st
7	6.46	0.51	7.11	0.45	t
8	6.16	0.46	7.96	0.78	t
9	6.11	0.24	24.38	0.87	sm/st
10	5.84	0.28	27.94	0.84	sm
11	5.83	0.43	8.10	0.64	t
12	5.52	0.29	24.75	0.82	sm/st
13	5.26	0.27	29.39	1.01	sm
14	5.13	0.23	21.76	0.91	St
15	4.74	0.31	22.02	0.61	St
16	4.49	0.33	30.43	0.76	Sm

m: metacentric, sm: submetacentric, st: subtelocentric; t: telocentric; sm/st: submeta/subtelocentric

Telomeric sequences were detected using a vertebrate telomeric (CCCTAA)_3 _PNA probe. Single distinct terminal signals appear at the ends of both sister chromatids of every mitotic chromosome in the two species (Figure 1d, h, l, p). No additional interstitial telomeric sequences were observed. Although the sizes of the telomeric signals do not show a high variability, consistently bigger signals appear at terminal regions of the long arm of one of the members of one chromosome pair in *Brachidontes puniceus *(Figure 1d) and of the short arms of two chromosome pairs in *B. rodriguezi *(Figure 1l). One of the *B. puniceus *mussels presents an additional bigger telomeric signal (Figure 1h) and one of the *B. rodriguezi *specimens shows only three bigger telomeric signals (Figure 1p).

DAPI/PI staining of mitotic metaphase plates reveals the presence of a DAPI negative (DAPI-) terminal region on the long arm of the subtelocentric chromosome pair # 15 in *Brachidontes puniceus *(Figure 1a, e). One of the mussels shows an additional DAPI- band on the short arm of the chromosome pair also bearing the standard band (Figure 1e). CMA staining of the same metaphase plates (Figure 1b, f) shows that these regions are CMA bright (CMA+). *B. rodriguezi *presents DAPI-/CMA+ regions on the short arms of the two telo/subtelocentric chromosome pairs, # 4 and # 6 (Figure 1i, j, m, n), but one of the individuals analyzed shows only three DAPI-/CMA+ positive regions in every single cell (Figure 1m, n).

FISH signals for the major ribosomal genes were analyzed in 130 complete metaphase plates in five *Brachidontes puniceus *mussels and in 150 metaphases in five specimens of *B. rodriguezi. B. puniceus *presents two major rDNA signals on a terminal region of the long arm of the subtelocentric chromosome pair # 15 (Figure 1c). These signals coincide with the DAPI-/CMA+ regions (Figure 1a, b). The mussel showing an additional DAPI-/CMA+ region (Figure 1e, f) also presents a third 28S rDNA signal at the same position (Figure 1g). Most *B. rodriguezi *mussels present four major ribosomal signals, coincident with the DAPI-/CMA+ regions, on the short arms of the telo/subtelocentric chromosome pairs # 4 and # 6 (Figure 1i, j, k), but the one showing only three DAPI-/CMA+ regions (Figure 1m, n) also presents only three major rDNA signals (Figure 1o). The consistently bigger telomeric signals appear at the DAPI-/CMA+ NORs.

FISH signals corresponding to the minor ribosomal genes were studied in 111 metaphases in *B. puniceus *and 150 in *B. rodriguezi*. As shown in Figure 2a, 5S rDNA signals are interstitially located on the long arms of chromosome pairs # 4 and # 6 in *B. puniceus*. In *B. rodriguezi *5S rDNA signals are also intercalary located on the long arms of two chromosome pairs, # 3 and # 4 (Figure 2b). The number of minor rDNA signals was always 4 in *B. puniceus *but 2 or 4 in different metaphase plates in *B. rodriguezi*. Double-color FISH experiments (Figure 2c, d) confirmed the presence of major and minor rDNA clusters on chromosome pair # 4 in *B. rodriguezi *and therefore allowed the study of the distribution of the variable number of 5S rDNA signals (Table 3). A 66.67% of the metaphase plates showed signals only on the NOR-bearing chromosome pair # 4. The remaining metaphases showed 4 signals on pairs # 3 and # 4 (32%), being always weaker those in chromosome # 3 (Figure 2b, d, h, j), or only 2 signals on pair # 3 (1.33%).

**Table 3:** Number and proportion of metaphases showing 2 or 4 5S rDNA signals on chromosome pairs # 3, not bearing 28S rDNA signals, and # 4, also bearing 28S rDNA signals, on five specimens of Brachidontes rodriguezi

Mussel	2 signals, # 3		2 signals, # 4		4 signals, # 3, # 4		Total
		
	n	%	n	%	n	%	
1	0	0.00	15	60.00	10	40.00	25
2	1	2.33	29	67.44	13	30.23	43
3	1	4.17	8	33.33	15	62.50	24
4	0	0.00	24	82.76	5	17.24	29
5	0	0.00	24	82.76	5	17.24	29

Total	2	1.33	100	66.67	48	32.00	150

Core histone genes were mapped by FISH using both histone H3 and histone H2B/H2A probes. FISH signals were analyzed in at least 20 complete metaphases per mussel in 5 individuals in each species. Core histone gene signals are interstitial to the long arms of chromosome pairs # 6 and # 10 in *B. puniceus *(Figure 2e). The signal in pair # 10 is closer to the centromere. *B. rodriguezi *also shows intercalary signals on two chromosome pairs, # 3 and # 9 (Figure 2f). In this species the signal on chromosome pair # 9 is closer to the centromere. Double-color FISH experiments using H3 and H2B/H2A histone gene probes labeled differently always showed coincident signals (not shown). In order to confirm the location of the histone gene clusters in relation to the minor and major rDNA clusters, double and triple-color FISH experiments were performed. One of the core histone gene clusters and one of the 5S rDNA clusters are on the same chromosome pair (Figure 2g, h) both in *B. puniceus *(pair # 6) and *B. rodriguezi *(# 3). The relative positions of the major rDNA, 5S rDNA and histone gene clusters are indicated on the karyotypes that appear on Figure 2i, j.

## Discussion

The chromosomal characterization of the mussels of the family Mytilidae includes the knowledge of mitotic chromosome numbers and karyotypes in 32 species. Six of these species show the same diploid chromosome number (2n = 32) present in both *Brachidontes puniceus *and *B. rodriguezi *[8]. On the contrary, the only other species of this genus in which the karyotype has been described, *B. pharaonis*, shows 2n = 28 chromosomes [10]. The variability of chromosome numbers between species of the same genus is widespread in the family Mytilidae; with the exception of *Mytilus*, all genera of mussels in which karyotypes have been determined for more than one species show differences in chromosome numbers [34]. On the other hand, the coincidence in chromosome numbers in *B. puniceus *and *B. rodriguezi *is accompanied by considerable differences in karyotype composition.

The detection of the vertebrate telomeric (TTAGGG)_n _repeat at chromosome ends in *Brachidontes puniceus *and *B. rodriguezi *agrees with results obtained in chromosomes of other species of bivalves [2], including the mussels *Mytilus galloprovincialis *[6,28] and *Perumytilus purpuratus *[5]. On the contrary, the interstitial telomeric sequences clearly detected by FISH in *P. purpuratus *[5] and presumably appearing in *M. galloprovincialis *[28] were not detected in these two species of *Brachidontes*. Although in bivalves telomeric sequences have only been isolated and characterized in *Donax trunculus *[28], the hybridization results obtained so far indicate that the bivalve telomeres are composed of tandem repeats of the hexanucleotide which also constitutes the vertebrate telomeric sequence.

The presence of major rDNA signals on CMA bright regions terminally located on a single chromosome pair in *Brachidontes puniceus *and on two chromosome pairs in *B. rodriguezi *is concordant with results obtained in other bivalves. NORs have been located in around 50 species of bivalves [2,5] and are located at terminal positions on one to three chromosome pairs. In the family Mytilidae, the position of major rRNA genes is known in seven species. One NOR-bearing chromosome pair has been detected in *B. pharaonis *[10], two in *Mytilus galloprovincialis *[6], *M. edulis *[11], *B. rodriguezi *[8] and *Perumytilus purpuratus *[5], and three in *M. trossulus *and *M. californianus *[9]. The species of the genus *Brachidontes *show differences both in number and chromosomal location of the NORs. The single major ribosomal gene cluster appears at a terminal position on the long arm of one small subtelocentric chromosome pair in both *B. puniceus *and *B. pharaonis *[10]. However, the two major ribosomal gene clusters of *B. rodriguezi *are spread over the whole short arms of the two subtelocentric/telocentric chromosomes pairs [8].

The occurrence of individuals showing a different number of rDNA clusters to those usually found in the species has been described in many taxa, including humans [35]. In this sense, the presence of an additional NOR in one of the *B. puniceus *specimens is not remarkable and could be the result of a translocation process as proposed for human ectopic NORs [35]. The absence of one of the major rDNA clusters in one chromosome in *B. rodriguezi *can be attributed to unequal crossover events [36].

The mostly coincident FISH signal sizes obtained at the terminal NORs after using 28S rDNA and telomeric probes in both *B. rodriguezi *and *B. puniceus *might indicate that some telomeric repeats are interspersed within the major rDNA repeats; fiber-FISH and molecular analysis are necessary to confirm or discard this hypothesis. Although not common, interspersion of telomeric and major ribosomal DNA sequences has been observed in bivalves, such as the mussel *Mytilus galloprovincialis *[6] and the scallop *Patinopecten yessoensis *[20], and in other organisms such as fishes [37] and mammals [38]. The meaning of such organization is not well understood but a functional role in nucleolus organization for tandem repeats has been proposed [39].

The chromosomal location of 5S rDNA clusters is only known in 15 species of bivalves [2,5]. In most of these species, minor rDNAs usually appear at interstitial loci [18-20]. The presence of two 5S rDNA clusters in both *Brachidontes puniceus *and *B. rodriguezi *differs from the results obtained in *Mytilus edulis *and *M. galloprovincialis *[11], in which four 5S rDNA clusters appear on three chromosome pairs, and *Perumytilus purpuratus *[5], showing three 5S rDNA clusters on two chromosome pairs. Regarding the other species of bivalves in which the location of these sequences is known, the Ostreidae [13,14] and two species of Pectinidae [15,17] also show two 5S rDNA clusters. On the contrary, *Cerastoderma edule *(Cardiidae) presents five clusters of 5S rDNA on five different chromosome pairs [12] and most species of Pectinidae show a single 5S rDNA cluster [16,18,20].

The variation in number of 5S rDNA FISH signals detected in *Brachidontes rodriguezi *is mostly due to the presence or absence of signals in chromosome pair # 3. The small size of these signals probably indicates that the number of 5S rDNA repeats at this locus is close to the minimum necessary for the signal to be detected. Similar variations in the number of 5S rDNA signals have been detected in two other species of Mytilidae, *Mytilus galloprovincialis *and *M. edulis *[11].

Our results demonstrate that major and minor rDNA clusters are on different chromosome pairs in *Brachidontes puniceus *but that in *B. rodriguezi *one of the NOR-bearing chromosome pairs also carries one of the 5S rDNA clusters. This is also the case in *Perumytilus purpuratus *[5] and *Chlamys farreri *[16] but differs from the presence of both major and 5S rDNA clusters on different chromosome pairs in the rest of the bivalve species studied so far.

Histone genes are usually organized as tandem repeats in invertebrate genomes [40]. These clusters can be composed by a copy of each one of the core histone genes or both core and linker histone genes. Histone gene arrangement in bivalves has been studied in the mussels *Mytilus edulis *[21,23] and *M. galloprovincialis *[22,24], and the scallop *Chlamys farreri *[25]. All of them present clusters of core histone genes ordered in the same way thus probably indicating a conserved arrangement. Our results showing hybridization signals at the same positions after FISH using H3 gene and H2B/H2A gene probes point also to this situation in *Brachidontes puniceus *and *B. rodriguezi*. Histone genes are known to be located in two chromosome pairs in the mussel *M. galloprovincialis *[24] and the scallop *Patinopecten yessoensis *[27] but forming a single cluster in other mussel species, *Perumytilus purpuratus *[5], and in the scallops *Argopecten irradians*, *Chlamys farreri *and *C. nobilis *[27]. Therefore, in the family Mytilidae, the presence of two histone gene clusters in these two *Brachidontes *species coincides with the situation in *M. galloprovincialis *[24] but differs from the one in *P. purpuratus *[5].

One of the histone gene clusters and one of the 5S rDNA clusters appear on the long arm of the same chromosome pair in both *Brachidontes puniceus *and *B. rodriguezi*. In addition, the relative positions of these clusters with respect to the centromere are similar in both species being the histone genes distal to the 5S rDNA. Although these facts seem to indicate that this chromosome is conserved, further analyses are necessary in order to determine their homology. The only other species of bivalves in which the position of these two sequences was investigated together is *Perumytilus purpuratus *[5]. In contrast with *B. puniceus *and *B. rodriguezi*, in *P. purpuratus *the single histone gene cluster and the 5S rDNA clusters are located in different chromosome pairs.

The similarities in the chromosomal distribution of major rDNA, 5S rDNA and core histone gene clusters found in *Brachidontes puniceus*, *B. rodriguezi *and *Perumytilus purpuratus *[5] are not shared by *Mytilus galloprovincialis *[11,22,24] and confirm the closeness of *P. purpuratus *to the species of *Brachidontes*, therefore keeping open the possibility of its assignation to this genus but discarding that *P. purpuratus*, *B. rodriguezi *and *B. darwinianus *belongs to the same taxon [41].

## Conclusions

The FISH results obtained in this work after using major rDNA, 5S rDNA and histone gene probes allow unequivocally identifying four of the 16 chromosome pairs that compose the karyotypes of *Brachidontes puniceus *and *B. rodriguezi*. Additional research is in progress in order to find more markers that allow chromosome identification in Mytilidae and therefore to gain insights on chromosome evolution in this family.

## Authors' contributions

CPG undertook most of the cytogenetic procedures and collaborated on the molecular work, the bibliographic review, and the writing of this paper. JGV participated in the first steps of the amplification, cloning, characterization and mapping of the 5S rDNA.

PM participated in developing the molecular techniques and helped in the writing. JJP coordinated the study, helped in developing the laboratory techniques and cytogenetic analyses and coordinated the writing of the manuscript. All authors read and approved the final manuscript.

## References

[B1] Giribet G Distel D Lydeard C, Lindberg DR Bivalve phylogeny and molecular data Systematics and Phylogeography of Mollusks 2003 Washington DC, Smithsonian Books 45 90

[B2] Leitão A Chaves R Russo R Banding for chromosomal identification in bivalves: A 20-year history Aquaculture 1. Dynamic Biochemistry, Process Biotechnology and Molecular Biology 2008 2 Special Issue 1 Global Science Books 44 49

[B3] Thiriot-Quiévreux C Review of the literature on bivalve cytogenetics in the last ten years Cah Biol Mar 2002 43 17 26

[B4] Guo X Wang Y Xu Z Liu Z Genomic analyses using fluorescence *in situ *hybridization Aquaculture genome technologies 2007 Oxford, Blackwell Publishing 289 311 full_text

[B5] Pérez-García C Cambeiro JM Morán P Pasantes JJ Chromosomal mapping of rDNAs, core histone genes and telomeric sequences in *Perumytilus purpuratus *(Bivalvia: Mytilidae) J Exp Mar Biol Ecol 2010 395 199 205

[B6] Martínez-Expósito MJ Méndez J Pasantes JJ Analysis of NORs and NOR-associated heterochomatin in the mussel *Mytillus galloprovincialis *Lmk Chromosome Res 1997 5 268 273 9244455 10.1023/a:1018475804613

[B7] Insua A Méndez J Physical mapping and activity of ribosomal RNA genes in mussel *Mytilus galloprovincialis* Hereditas 1998 128 189 194 10.1111/j.1601-5223.1998.00189.x 9760868

[B8] Torreiro A Martínez-Expósito MJ Trucco MI Pasantes JJ Cytogenetics in *Brachidontes rodriguezi *d'Orb (Bivalvia, Mytilidae) Chromosome Res 1999 7 49 55 10.1023/A:1009275311888 10219732

[B9] González-Tizón AM Martínez-Lage A Rego I Ausio J Méndez J DNA content, karyotypes, and chromosomal location of 18S-5.8S-28S ribosomal loci in some species of bivalve molluscs from the Pacific Canadian coast Genome 2000 43 1065 1072 11195339 10.1139/g00-089

[B10] Vitturi R Gianguzza P Colomba MS Riggio S Cytogenetic characterization of *Brachidontes pharaonis *(Fisher P, 1870): Karyotype, banding and fluorescent *in situ *hybridization (FISH) (Mollusca: Bivalvia: Mytilidae) Ophelia 2000 52 213 220

[B11] Insua A Freire R Ríos R Méndez J The 5S rDNA of mussels *Mytilus galloprovincialis *and *M. edulis*: sequence, variation and chromosomal location Chromosome Res 2001 9 495 505 10.1023/A:1011636714052 11592484

[B12] Insua A Freire R Méndez J The 5S rDNA of the bivalve *Cerastoderma edule*: nucleotide sequence of the repeat unit and chromosomal location relative to 18-28S rDNA Genet Sel Evol 1999 31 509 518 10.1186/1297-9686-31-5-509

[B13] Cross I Díaz E Sánchez I Rebordinos L Molecular and cytogenetic characterization of *Crassostrea angulata *chromosomes Aquaculture 2005 247 135 144 10.1016/j.aquaculture.2005.02.039

[B14] Wang Y Xu Z Guo X Chromosomal mapping of 5S ribosomal RNA genes in the eastern oyster, *Crassostrea virginica *Gmelin by fluorescence *in situ *hybridization J Shellfish Res 2005 24 959 964

[B15] Insua A López-Piñón MJ Méndez J Characterization of *Aequipecten opercularis *(Bivalvia: Pectinidae) chromosomes by different staining techniques and fluorescent *in situ *hybridization Genes Genet Syst 1998 73 193 200 10.1266/ggs.73.193 9880917

[B16] Wang Y Guo X Chromosomal rearrangement in Pectinidae revealed by rRNA loci and implications for bivalve evolution Biol Bull 2004 207 247 256 10.2307/1543213 15616355

[B17] López-Piñón MJ Insua A Méndez J Chromosome analysis and mapping of ribosomal genes by one and two-color fluorescent *in situ *hybridization in *Hinnites distortus *(Bivalvia: Pectinidae) J Heredity 2005 96 52 58 10.1093/jhered/esi001 15598716

[B18] Insua A López-Piñón MJ Freire R Méndez J Karyotype and chromosomal location of 18S-28S and 5S ribosomal DNA in the scallops *Pecten maximus *and *Mimachlamys varia *(Bivalvia: Pectinidae) Genetica 2006 126 291 301 10.1007/s10709-005-7408-7 16636923

[B19] Huang X Hu J Hu X Zhang G Zhang L Wang S Lu W Bao Z Cytogenetic characterization of the bay scallop, *Argopecten irradians irradians*, by multiple staining techniques and fluorescence *in situ *hybridization Genes Genet Syst 2007 82 257 263 10.1266/ggs.82.257 17660696

[B20] Huang X Hu X Hu J Zhang L Wang S Lu W Bao Z Mapping of ribosomal DNA and (TTAGGG)n telomeric sequence by FISH in the bivalve *Patinopecten yessoensis *(Jay, 1857) J Moll Stud 2007 73 393 398 10.1093/mollus/eym036

[B21] Drabent B Kim JS Albig W Prats E Cornudella L Doenecke D *Mytilus edulis *histone gene clusters containing only H1 genes J Mol Evol 1999 49 645 655 10.1007/PL00006585 10552045

[B22] Eirín-López JM González-Tizón AM Martínez A Méndez J Molecular and evolutionary analysis of mussel histone genes (*Mytilus *spp): possible evidence of an "orphon origin" for H1 histone genes J Mol Evol 2002 55 272 283 12187381 10.1007/s00239-002-2325-1

[B23] Albig W Warthorst U Drabent B Prats E Cornudella L Doenecke D *Mytilus edulis *core histone genes are organized in two clusters devoid of linker histone genes J Mol Evol 2003 56 597 606 10.1007/s00239-002-2428-8 12698296

[B24] Eirín-López JM Ruiz MF González-Tizón AM Martínez A Sánchez L Méndez J Molecular evolutionary characterization of the mussel *Mytilus *histone multigene family: first record of a tandemly repeated unit of a five histone genes containing an H1 subtype whit "orphon" features J Mol Evol 2004 58 131 144 15042333 10.1007/s00239-003-2531-5

[B25] Li C Song L Zhao J Zou H Su J Zhang H Genomic organization, nucleotide sequence analysis of the core histone genes cluster in *Chlamys farreri *and molecular evolution assessment of the H2A and H2B DNA Seq 2006 17 440 445 17381045 10.1080/10425170600752593

[B26] González-Romero R Ausió J Méndez J Eirín-López JM Early evolution of histone genes: Prevalence of an 'orphon' H1 lineage in Protostomes and birth-and-death process in the H2A family J Mol Evol 2008 66 505 518 18443735 10.1007/s00239-008-9109-1

[B27] Zhang L Bao Z Wang S Huang X Hu J Chromosome rearrangements in Pectinidae (Bivalvia: Pteriomorphia) implied based on chromosomal localization of histone H3 gene in four scallops Genetica 2007 130 193 198 10.1007/s10709-006-9006-8 16909332

[B28] Plohl M Prats E Martínez-Lage E González-Tizón A Méndez J Cornudella L Telomeric localization of the vertebrate-type hexamer repeat, (TTAGGG)n, in the wedgeshell clam *Donax trunculus *and other marine invertebrate genomes J Biol Chem 2002 277 19839 19846 10.1074/jbc.M201032200 11907038

[B29] Levan A Fredga K Sandberg AA Nomenclature for centromeric position on chromosomes Hereditas 1964 52 201 220 10.1111/j.1601-5223.1964.tb01953.x

[B30] Estoup A Largiadèr CR Perrot E Chourrout D Rapid one-tube DNA extraction for reliable PCR detection of fish polymorphic markers and transgenes Mol Mar Biol Biotechnol 1996 5 295 298

[B31] White TJ Burms T Lee S Taylor JW Inmus MA, Guelfand DH, Sminsky JJ, White TJ Amplification and direct sequences of fungal ribosomal RNA genes for phylogenetics PCR protocols: a guide to methods and applications 1990 New York, Academic Press 315 322

[B32] Fang BL De Baere R Vandenberghe A De Wachter R Sequences of three molluscan 5S ribosomal RNAs confirm the validity of a dynamic secondary structure model Nucleic Acids Res 1982 10 4679 4685 10.1093/nar/10.15.4679 7133995 PMC321121

[B33] Hurtado NS Pasantes JJ Surface spreading of synaptonemal complexes in the clam *Dosinia exoleta *(Mollusca, Bivalvia) Chromosome Res 2005 13 575 580 10.1007/s10577-005-0983-8 16170622

[B34] Thiriot-Quiévreux C Beaumont AR Advances in cytogenetics of aquatic organisms Genetics and Evolution of Aquatic Organisms 1994 London, Chapman & Hall 369 388

[B35] Kühl H Rötger S Heilbronner H Enders H Schempp W Loss of Y chromosomal PAR2-region in four familial cases of satellite Y chromosomes (Yqs) Chromosome Res 2001 9 215 222 11330396 10.1023/a:1012219820317

[B36] Deiana AM Cau A Salvadori S Coluccia E Cannas R Milia A Tagliavini J Major and 5S ribosomal sequences of the largemouth bass *Micropterus salmoides *(Perciformes, Centrarchidae) are localized in GC-rich regions of the genome Chromosome Res 2000 8 213 218 10.1023/A:1009248828365 10841048

[B37] Abuín M Martínez P Sánchez L Localization of repetitive telomeric sequence (TTAGGG)n in four salmonid species Genome 1996 39 1035 1038 8890525 10.1139/g96-129

[B38] Zhdanova NS Minina JM Karamisheva TV Draskovic I Rubtsov NB Londoño-Vallejo JA The very long telomeres in *Sorex granarius *(Soricidae, Eulipothyphla) contain ribosomal DNA Chromosome Res 2007 15 881 890 10.1007/s10577-007-1170-x 17899406

[B39] Kaplan FS Murray J Sylvester JE Gonzalez IL O'Connor P Doering JL Muenke M Emanuel BS Zasloff MA The topographic organization of repetitive DNA in the human nucleolus Genomics 1993 15 123 132 10.1006/geno.1993.1020 8432523

[B40] Eirín-López JM González-Romero R Dryhurst D Méndez J Ausió J Pontarotti P Long-term evolution of histone families: old notions and new insights into their mechanisms of diversification across eukaryotes Evolutionary Biology 2009 Berlin, Springer-Verlag 139 16219549324

[B41] Aguirre ML Pérez SI Sirch YN Morphological variability of *Brachidontes *Swainson (Bivalvia, Mytilidae) in the marine Quaternary of Argentina (SW Atlantic) Paleogeogr Paleoclimatol Paleoecol 2006 239 100 125 10.1016/j.palaeo.2006.01.019

